# Closure of Petersen’s defect in gastrectomy for gastric cancer: an interrupted time series analysis from a high-volume institution in China

**DOI:** 10.1007/s00423-020-02019-2

**Published:** 2020-11-05

**Authors:** Tao Pan, Hui Wang, Kai Liu, Xin-zu Chen, Wei-han Zhang, Xiao-long Chen, Kun Yang, Bo Zhang, Zong-guang Zhou, Jian-kun Hu

**Affiliations:** Department of Gastrointestinal Surgery and Laboratory of Gastric Cancer, State Key Laboratory of Biotherapy, West China Hospital, Sichuan University and Collaborative Innovation Center for Biotherapy, No. 37 GuoXue Xiang Street, Chengdu, 610041 Sichuan Province China

**Keywords:** Gastric cancer, Petersen’s hernia, Gastrectomy

## Abstract

**Purpose:**

Petersen’s hernia (PH) is a serious complication after gastrectomy for gastric cancer. The aim of this study was to investigate whether closure of Petersen’s defect (PD) can decrease the rates of PH and suspected Petersen’s hernia (SPH).

**Methods:**

Patients who underwent gastrectomy with PD were enrolled. From January 2014 to January 2017, we performed gastrectomy without PD closure (non-closure group). From February 2017 to June 2018, we closed PDs during gastrectomy (closure group). The rates of PH and SPH were compared between the two groups. The last follow-up was updated in August 2020.

**Results:**

Among a total of 1213 patients, 12 patients (1.0%) developed PH, and 23 patients (1.9%) developed SPH. The rate of PH in the closure group was significantly lower than that in the non-closure group (1/385, 0.3% versus 11/828, 1.3%, *p* = 0.042, log-rank test). The rate of SPH in the closure group was significantly lower than that in the non-closure group (1/385, 0.3% versus 22/828, 2.7%, *p* = 0.008, log-rank test). Non-closure of PD was a risk factor for PH and SPH (odds ratio (OR) 7.72, 95% CI 1.84–32.35, *p* = 0.006).

**Conclusions:**

PD closure is recommended after gastrectomy for gastric cancer, as the rates of PH and SPH were significantly decreased.

## Introduction

An internal hernia can lead to small bowel obstruction and life-threatening conditions, such as bowel ischemia or perforation [1, 2]. Internal hernia is a recognized and well-described complication after laparoscopic Roux-Y gastric bypass [3–6]. However, there have been few studies about internal hernia after gastrectomy for gastric cancer [1, 7], and to our knowledge, there has been no study on Petersen’s hernia (PH) after gastrectomy for gastric cancer.

The low incidence and nonspecific manifestations of PH make it difficult to diagnose preoperatively [7–9]. Some non-operatively managed patients are highly suspicious for PH according to manifestations and computed tomography (CT) scans [10, 11]; however, some of these patients might be misdiagnosed as having adhesive small bowel obstructions and managed non-operatively [12, 13]. Patients with suspected Petersen’s hernia (SPH) may have nonspecific and recurrent abdominal pain; they are also at risk of incarcerated internal hernia and bowel necrosis; therefore, great attention should be paid to these patients.

Closure of Petersen’s defect (PD) is now recommended for laparoscopic Roux-Y gastric bypass [2, 5]. However, PH can occur despite the closure of PDs [14]. There is also concern that closure by itself may increase the risk of bleeding, mesenteric hematoma, and anastomotic leakage due to vascular injury [3]. PDs are also created in Billroth-2 (B-2) and Roux-en-Y (R-Y) reconstructions after gastrectomy for gastric cancer. However, to date, there has been no consensus on the management of PD after gastrectomy for gastric cancer.

Therefore, the purpose of our study was to investigate whether PD closure during gastrectomy can decrease the rates of PH and SPH. To our knowledge, this was the first study to specifically investigate the rate of PH following gastrectomy for gastric cancer. We also included SPH as an endpoint in this study.

## Materials and methods

### Ethical statement

The study was based on information collected from the Surgical Gastric Cancer Patient Registry of West China Hospital (WCH-SGCPR-2019-10). The establishment of the database was approved by the Research Ethics Committee of West China Hospital. Additionally, because this was an interrupted time series study [15], patients did not provide written informed consent, but personal information was anonymized before analysis.

### Patients

**Fig. 1:**
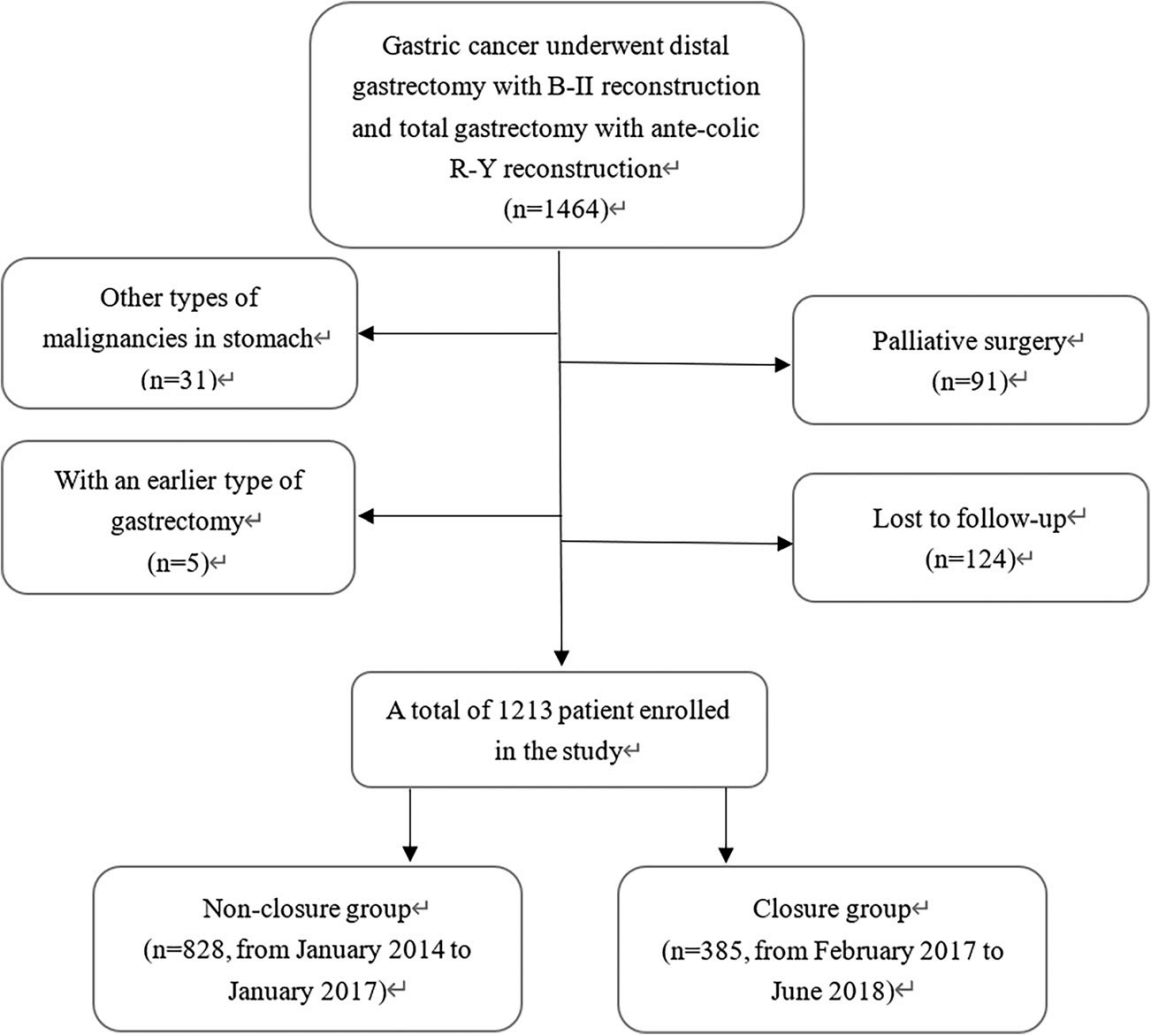
The flowchart of patients enrolled in the study

A total of 1213 consecutive patients with gastric cancer treated from January 2014 to June 2018 in West China Hospital were eligible for the study. The diagnosis of gastric cancer was confirmed in all patients by upper gastrointestinal endoscopy and biopsy. The inclusion criteria were as follows: (1) patients with histologically proven gastric adenocarcinomas and (2) patients who underwent curative distal gastrectomy with B-2 reconstruction and total gastrectomy with ante-colic R-Y reconstruction. The exclusion criteria were as follows: (1) other types of malignancies of the stomach; (2) palliative surgery; (3) previous history of gastrectomy; (4) lost to follow-up. A flowchart of the patients enrolled in the study is shown in Fig. 1.

### Clinicopathological materials

Clinicopathological data including sex, age, body mass index (BMI), previous abdominal surgery, surgical approach, reconstruction type (distal gastrectomy with B-2 anastomosis or total gastrectomy with ante-colic R-Y anastomosis), extent of lymphadenectomy, tumor diameter, tumor location, macroscopic type, tumor differentiation, pathologic TNM stage, and postoperative adjuvant chemotherapy were evaluated. All definitions, including the macroscopic type, tumor differentiation, and TNM stage, were determined according to the 7th Staging Manual of the American Joint Committee on Cancer [16].

### Surgical technique

**Fig. 2:**
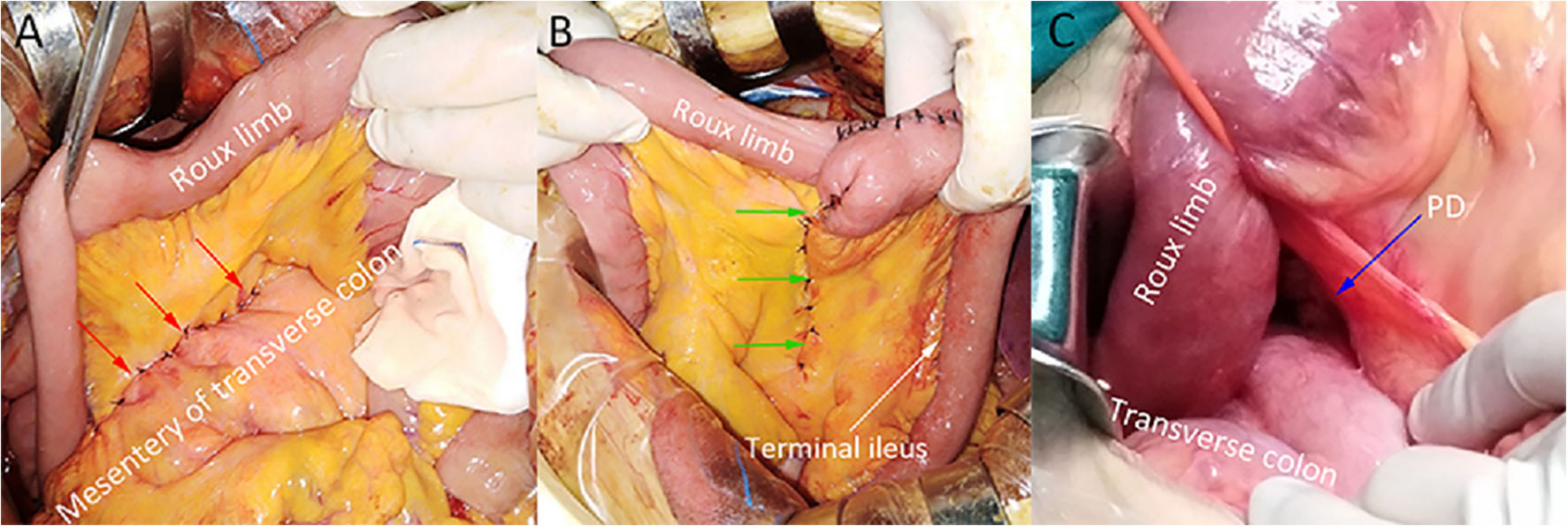
Operative pictures. **a** Closure of Petersen’s defect (PD, red arrows). **b** Closure of jejunojejunostomy mesenteric defect (green arrows). **c** PD (blue arrow) was found open after reduction of the Petersen’s hernia (PH)

The surgical treatment principles were based on the Japanese Gastric Cancer Treatment Guidelines [17, 18]. For reconstruction, B-2 reconstruction was adopted for distal gastrectomy and ante-colic R-Y reconstruction was adopted for total gastrectomy. Jejunojejunostomy mesenteric defects were all closed for patients who underwent R-Y reconstruction in both groups. From January 2014 to January 2017, we performed R-Y and B-2 reconstruction without closure of PDs (non-closure group). From February 2017 to June 2018, we closed PDs for both R-Y and B-2 reconstruction (closure group). We closed all defects using an interrupted 3-0 non-absorbable suture (Fig. 2a, b).

### Follow-up

It was recommended that all patients be examined every 3 months in the first 2 years, every 6 months in the third year, and at least once a year in the following years. A complete blood count and chemistry file were performed during every follow-up. CT examination of the chest, abdomen, and pelvis was performed every 6 months in the first 3 years and at least once a year thereafter. Upper endoscopy was performed every 6 months for the first year, annually from the second to the fifth year, and as clinically indicated thereafter. However, in emergency situations (such as acute abdominal pain), we recommend that the patient be examined and treated at the hospital immediately.

Among all the patients in the present study, 985 (77.9%) patients had examinations in our hospital. These patients went to our outpatient clinic after the examination. The outpatient doctors recorded the results of the abnormal tests and updated them in our gastric cancer database. Details of these examinations can be also obtained through our electronic system when needed. In our present study, 268 (22.1%) patients had examinations in other hospitals. Our doctors and members of the Volunteer Team of Gastric Cancer Surgery in our hospital made a telephone call to all the patients every 6 months. So, for all the patients, their records can be updated at least twice a year. Postoperative follow-up was mainly performed either at the postoperative outpatient clinic or by telephone. In recent years, we have started using WeChat as a supplement. We asked all our patients or their families to join our patient contact group on WeChat after gastrectomy. For those who had examinations in other hospitals and could not provide accurate information by telephone, we asked them to come to our outpatient clinic or upload their inpatient records and examinations through our patient contact group on WeChat.

Follow-up information was also collected from the database and updated on August 1, 2020. The duration of follow-up was recorded as the time from gastrectomy until death or the last registered follow-up at the postoperative outpatient clinic or by telephone, whichever came first.

### Endpoints

The primary endpoints were PH and SPH. The secondary endpoints were postoperative complications within 30 days of gastrectomy.

PH was defined as an internal hernia located at the PD and was confirmed by surgical exploration.

**Fig. 3:**
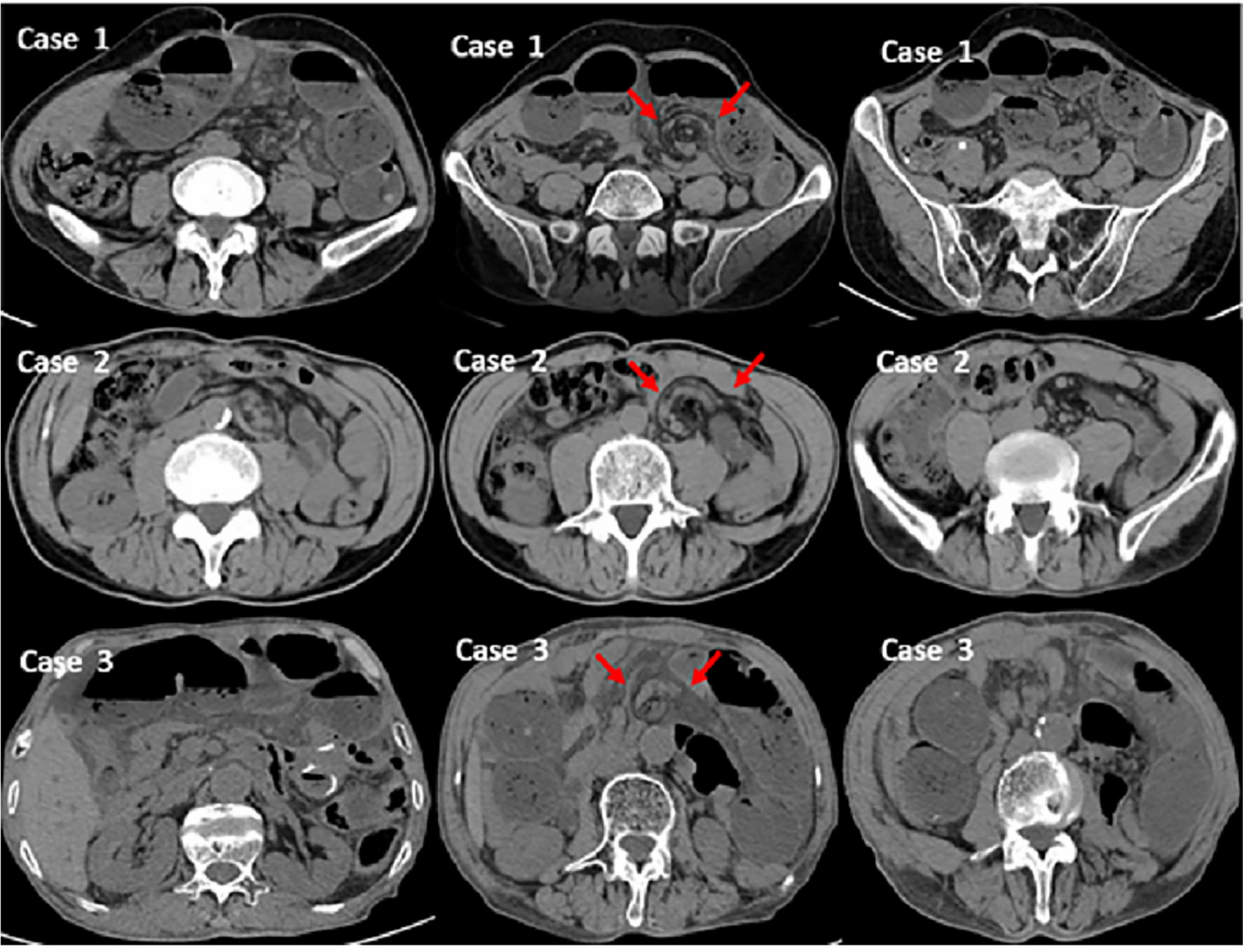
Whirling appearance of the mesentery and mesenteric vessels on CT scans (“whirl sign”, red arrows)

SPH was defined as patients who showed small bowel obstruction and a whirling appearance of the mesentery on CT scans (“whirl sign”, Fig. 3) after gastric cancer surgery. These patients were not confirmed by surgery. The “whirl sign” on the CT scan was confirmed by two radiologists and was described in previous studies [10, 11, 19–21].

Early postoperative complications were classified according to the Clavien-Dindo surgical complication grading system [22]. When a patient had two or more postoperative complications, the higher grade was recorded [23].

### Statistical analysis

Categorical variables are presented as numbers with percentages, and continuous data are presented as means with the standard deviation (SD). Categorical data were compared with the Pearson *χ*^2^ test or Fisher’s exact test, and continuous data were compared with an independent samples *t* test or a Wilcoxon rank-sum test as appropriate. The rates of PH and SPH were compared between the two groups by a log-rank test. A logistic regression analysis was performed to test the univariate and multivariate associations between variables to identify risk factors for PH and SPH. A two-sided value of *p* < 0.050 was considered significant. Data were analyzed using SPSS 20.0 statistical software (SPSS®, Chicago, IL, USA).

## Results

### Baseline data

A total of 1213 patients were enrolled in our study. The non-closure group included 828 patients, and the closure group included 385 patients. The demographic data of the entire patient population are presented in Table 1. There were no significant differences between the two groups with respect to sex, age, BMI, previous abdominal surgery, surgical approach, reconstruction type, extent of lymphadenectomy, tumor diameter, tumor location, macroscopic type, tumor differentiation, TNM stage, or postoperative adjuvant chemotherapy.

**Table 1:** Demographic data at the time of gastrectomy

	Non-closure group (first period, *N* = 828)	Closure group (second period, *N* = 385)	*p*†
Sex			0.077
Male	592 (71.5%)	256 (66.5%)	
Female	236 (28.5%)	129 (33.5%)	
Mean age at operation (years)*	58.0 ± 11.4	59.0 ± 11.1	0.077‡
Body mass index (kg/m^2^)*	22.6 ± 2.9	22.9 ± 3.2	0.089‡
Previous abdominal surgery			0.092
Yes	137 (16.5%)	79 (20.5%)	
No	691 (83.5%)	306 (79.5%)	
Surgical approach			0.516
Open	721 (87.1%)	330 (85.7%)	
Laparoscopy assisted	107 (12.9%)	55 (14.3%)	
Reconstruction type			
B-2 (distal gastrectomy)	551 (66.5%)	257 (66.8%)	0.943
R-Y (total gastrectomy)	277 (33.5%)	128 (33.2%)	
Extent of lymphadenectomy			0.916
D1/D1+	157 (19.0%)	73 (19.0%)	
D2/D2+	671 (81.0%)	312 (81.0%)	
Tumor diameter (cm)*	4.6 ± 2.5	4.4 ± 2.5	0.208‡
Tumor location			0.813
Upper	180 (21.7%)	87 (22.6%)	
Middle	118 (14.3%)	59 (15.3%)	
Lower	516 (62.3%)	231 (60.0%)	
Whole	14 (1.7%)	8 (2.1%)	
Macroscopic type			
Early gastric cancer	182 (22.0%)	85 (22.1%)	0.761
Borrmann-1	17 (2.1%)	7 (1.8%)	
Borrmann-2	298 (36.0%)	140 (36.4%)	
Borrmann-3	282 (34.1%)	131 (34.0%)	
Borrmann-4	49 (5.9%)	22 (5.7%)	
Tumor differentiation			0.307
Well/moderately	226 (27.3%)	116 (30.1%)	
Poorly/undifferentiated	602 (72.7%)	269 (69.9%)	
Pathological TNM stage			0.568
I	217 (26.2%)	102 (26.5%)	
II	213 (25.7%)	109 (28.3%)	
III	398 (48.1%)	174 (45.2%)	
Postoperative adjuvant chemotherapy			0.649
Yes	507 (61.2%)	241 (62.6%)	
No	321 (38.8%)	144 (37.4%)	

Values in parentheses are percentages unless indicated otherwise

*Values are mean ± standard deviation. *R-Y*, Roux-en-Y reconstruction; *B-2*, Billroth-2 reconstruction

†*χ*^2^ test, except ‡ paired *t* test

### Early postoperative complications

Table 2 shows the detailed information of complications occurring within 30 days of gastrectomy. There was no significant difference between the two groups with respect to postoperative complications, non-surgical complications, or surgical complications (*p* = 0.457, 0.571, and 0.106, respectively).

**Table 2:** Detailed information about complications within 30 days of gastrectomy

	Non-closure group (first period)	Closure group (second period)	*p*⇞
Postoperative complications (total)^**⊰**^	132 (15.9%)	55 (14.3%)	0.457
Non-surgical complications	96 (11.6%)	49 (12.7%)	0.571
Pulmonary disease	88 (10.6%)	43 (11.2%)	
Urinary disease	5 (0.6%)	3 (0.8%)	
Cardiac disease	0	1 (0.3%)	
Delirium	2 (0.2%)	1 (0.3%)	
Venous thromboembolism	0	1 (0.3%)	
Liver dysfunction	1 (0.1%)	0	
Surgical complications	43 (5.2%)	12 (3.1%)	0.106
Anastomotic/stump leakage	5 (0.6%)^∝^	1 (0.3%)^#^	
Surgical site infection	10 (1.4%)	3 (0.8%)	
Pancreatic fistula	3 (0.4%)	1 (0.3%)	
Lymphatic leakage	1 (0.1%)	0	
Postoperative bleeding	4 (0.5%)^¶^	3 (0.8%)	
Delayed gastric emptying	19 (2.3%)	4 (1.0%)	
Other major complications	1 (0.1%)^§^	0	
Clavien-Dindo classification			0.245
I–II	125 (15.1%)	52 (13.5%)	
III–V	6 (0.8%)^★^	3 (0.8%)	

Values in parentheses are percentages

^**⊰**^7 patients in the non-closure group and 6 patients in the closure group had both surgical and general complications

^∝^Site of leakage was gastrojejunostomy for 1 patient and duodenal stump for 4 patients

^#^Site of leakage was duodenal stump for 1 patient

^¶^Site of bleeding was the abdominal cavity for 3 patients and gastrointestinal tract for 1 patient

Site of bleeding was the abdominal cavity for 2 patient and the gastrointestinal tract for 1 patient

^§^Small bowel perforation due to gallstone

^★^1 patient died of pulmonary infection and respiratory failure

⇞*χ*^2^ test

### Rates of PH and SPH

Among a total of 1213 patients, 12 patients (1.0%) developed PH, and 23 patients (1.9%) developed SPH. In the non-closure group, which included 828 patients, 11 patients (1.3%) developed PH, and 22 patients (2.6%) developed SPH. The mean time intervals of PH and SPH were 14.4 and 11.3 months, respectively. After routine PD closure in 385 patients in the closure group, one patient (0.3%) developed PH after 22 months, and one patient (0.3%) developed SPH after 19 months. The rates of PH and SPH in the closure group were significantly lower than those in the non-closure group (*p* = 0.042 and 0.009, respectively, log-rank test).

### Characteristics of patients with PH

Table 3 shows the characteristics of patients with PH. Eleven patients in the non-closure group and one patient in the closure group developed PH. The PD was found to be open during the operation (Fig. 1c). We closed all PDs after the reduction of the herniated bowels, and no PH recurrence was observed until the end of the study. All 12 patients showed “whirl signs” on CT scans preoperatively. Among them, 10 patients underwent emergency surgery, and two patients underwent elective surgery. The two patients in the elective surgery group were diagnosed with SPH before surgery according to previous diagnostic criteria [10, 11, 19–21], and they were confirmed to have PH by surgical exploration.

**Table 3:** Characteristics of patients with PH

Case	Age	Sex	Interval period (months)	Whirl sign	Closure of PD	Procedure of gastrectomy	Bowel resection	Type of surgery	Mortality
1	58	Male	22	Yes	Yes	Laparoscopy-assisted distal gastrectomy	No	Emergency	No
2	65	Male	24	Yes	No	Open distal gastrectomy	No	Emergency	No
3	64	Male	7	Yes	No	Open distal gastrectomy	No	Emergency	Yes*
4	47	Female	1	Yes	No	Laparoscopy-assisted distal gastrectomy	No	Elective	No
5	44	Female	7	Yes	No	Open total gastrectomy	Yes	Emergency	No
6	79	Male	27	Yes	No	Open total gastrectomy	No	Emergency	No
7	67	Male	16	Yes	No	Open distal gastrectomy	No	Emergency	No
8	78	Male	6	Yes	No	Open distal gastrectomy	No	Emergency	No
9	66	Male	1	Yes	No	Open distal gastrectomy	Yes	Emergency	No
10	60	Female	7	Yes	No	Open distal gastrectomy	No	Elective	No
11	47	Female	28	Yes	No	Laparoscopy-assisted total gastrectomy	No	Emergency	No
12	50	Female	27	Yes	No	Open distal gastrectomy	Yes	Emergency	No

*Died of sepsis caused by bowel necrosis; *PH*, Petersen’s hernia; *BMI*, body mass index; *PD*, Petersen’s defect

### Risk factors for PH and SPH

Table 4 shows the results of univariate and multivariate analyses to identify the independent risk factors for PH and SPH. In the multivariate analysis, non-closure of the PD was the only risk factor for PH and SPH (odds ratio (OR) 7.72, 95% CI 1.84–32.35, *p* = 0.006). Sex, age, BMI, previous abdominal surgery, surgical approach, reconstruction type, and the extent of lymphadenectomy were not associated with PH and SPH occurrence.

**Table 4:** Results of univariate and multivariate analyses to identify the independent risk factors for PH and SPH

	PH and SPH		Univariate analysis		Multivariate analysis	
	(No, *n* = 1178)	(Yes, *n* = 35)	OR (95% CI)	*p*	OR (95% CI)	*p*
Sex						
Female	355	10	1.00 (reference)			
Male	823	25	1.08 (0.51–2.27)	0.842		
Age (years)						
≥ 65	367	12	1.00 (reference)			
< 65	811	23	0.87 (0.42–1.76)	0.694		
BMI (kg/m^2^)						
≥ 25	925	27	1.00 (reference)			
< 25	253	8	1.08 (0.49–2.41)	0.845		
Previous abdominal surgery						
Yes	214	2	1.00 (reference)		1.00 (reference)	
No	964	33	3.66 (0.87–15.38)	0.076	3.43 (0.81–14.45)	0.093
Surgical approach						
Laparoscopy assisted/robot	160	2	1.00 (reference)			
Open	1018	33	2.59 (0.62–10.91)	0.194		
Type of reconstruction						
R-Y (total gastrectomy)	391	14	1.00 (reference)			
B-2 (distal gastrectomy)	787	21	0.75 (0.38–1.48)	0.402		
Extent of lymphadenectomy						
D1/D1+	222	8	1.00 (reference)			
D2/D2+	956	27	0.79 (0.45–6.18)	0.417		
Closure of PD						
Yes (second period)	383	2	1.00 (reference)		1.00 (reference)	
No (first period)	795	33	7.95 (1.90–33.30)	0.005*	7.72 (1.84–32.35)	0.006*

Values in parentheses are 95% confidence intervals. *OR*, odds ratio; *CI*, confidence interval; *PH*, Petersen’s hernia; *SPH*, suspected Petersen’s hernia; *BMI*, body mass index; *R-Y*, Roux-en-Y reconstruction; *B-2*, Billroth-2 reconstruction; *PD*, Petersen’s defect

*Statistically significant difference (*p* < 0.05)

## Discussion

Gastric cancer is a major health problem, as it is the second leading cause of cancer death and the fourth most common cancer worldwide [24]. Surgery is a major curative strategy for gastric cancer [24]. A PD is created after gastrectomy with B-2 or R-Y reconstruction. However, there has been no consensus yet on how to deal with it.

The study showed that the rates of PH and SPH were significantly decreased after PD closure. The analysis of the risk factors for PH and SPH additionally validated these findings. Our results were consistent with those of previous studies [8, 21, 25].

Theoretically, if PDs are completely closed, no PH can occur. However, similar to a previous study [14], one case of PH occurred after we began closing PDs. We found that the PH patient underwent gastrectomy during the first week when we started to close PDs, and there have been no PH patients in the closure group since then. Both surgeons participating in the study were very well experienced with gastrectomy and far beyond their learning curve for this operation, but this was not necessarily the case for PD closure. Therefore, the reason for this case of PH may be incomplete closure of the PD during primary surgery [26]. Another explanation is that defects may open after the loss of mesenteric fat, leading to the formation of PH [27]. Therefore, although the closure of all mesenteric defects cannot completely prevent PH, current studies have shown that it may decrease the incidence rate.

To our knowledge, there has been no study that specifically investigated the rate of PH after gastrectomy for gastric cancer. Several studies examining internal hernias after gastrectomy for gastric cancer have been conducted. The overall rate of internal hernia (they were all PDs in this study) was 1.0% in our study, and the rate ranged from 0.19–5.0% in previous studies [1, 7, 14, 21]. The rate of internal hernia varied greatly among different studies. These differences may be caused by different inclusion groups, diagnostic criteria, follow-up periods, laparoscopy proportions, and mesenteric defect closures [1]. The rate of internal hernia in this study was lower than that in most previous studies. The possible reason is that we routinely closed the jejunojejunostomy mesenteric defects in all patients, and no internal hernia was found at these defects in this study; however, most authors left the defects open before they changed their technique to close all mesenteric defects, and many internal hernias were located at jejunojejunostomy mesenteric defects in their studies. In a study conducted by Miyagaki et al. [14], all gastrectomies, regardless of reconstruction method or gastrectomy type, were investigated, including patients with little possibility of internal hernia such as those who underwent esophagogastrostomy and Billroth-1 reconstruction. The 3-year incidence rate of internal hernia in their study was 0.19%, which was the lowest in literature.

In previous studies, laparoscopic surgery was found to be a risk factor for PH [1, 7, 14, 21, 28]. The possible reason was fewer adhesions [1, 14]. However, similar to a previous study [8], laparoscopic surgery was not a risk factor for PH in the present study. A possible explanation was that we adopted laparoscopy-assisted surgery in most cases. However, most authors mainly adopted total laparoscopic surgery in previous studies. Laparoscopy-assisted surgery may result in more adhesions than total laparoscopic surgery.

In previous studies on the effect of the closure of mesenteric defects after gastrectomy for gastric cancer, the internal hernia was defined as the only endpoint, but if suspected internal hernias were not included, the effect of mesenteric defect closure could have been overlooked. Due to its rare incidence and nonspecific symptoms, it is difficult to diagnose PH preoperatively. CT scans have become the main method to diagnose PH preoperatively [10], and some authors suggest that the most predictive signs of PH included the “whirl sign” and small bowel obstruction on CT scans (sensitivity 78–100%, specificity 80–90%) [3, 11, 19, 20]. Kang et al. [21] even used the “whirl sign” as a diagnostic criterion for internal hernia in their study. In the present study, 2 patients were considered to have SPH according to previous diagnostic criteria [10, 11, 19–21] before surgery, and they were finally confirmed to be PH by surgical exploration. Following the same diagnostic criteria, there were 23 SPH patients in our study. There is a strong possibility that some of these patients had PH, although they were not confirmed by surgery. These patients were managed non-operatively because they had no signs of bowel necrosis, and they were reluctant to receive surgery, or surgery was not physically possible.

There is concern that closure of mesenteric defects may be associated with a higher rate of postoperative complications such as mesenteric hematomas and bleeding. However, in our study, there was no difference in the rate of complications within 30 days between the non-closure and closure groups, indicating that PD closure did not increase early postoperative complications.

The strengths of the study were that it followed a standardized surgical protocol and included two distinct groups for comparison. Both surgeons participating in the study were well and equally experienced in gastrectomy for gastric cancer. The limitations of the study include that it was a retrospective study conducted in a single center. Another potential limitation of the study was that the follow-up duration was different between the two groups. However, the rate of PH seems to be highest within 1–2 years after operation [29–31], corresponding to the time of the greatest weight loss [3]. All the patients in the closure group were followed up for at least 26 months or until death, we would not expect a large number of additional PHs in this group. A multicenter prospective study is required to evaluate patients with the closure of all mesenteric defects during gastrectomy, including postoperative complications and quality of life.

In conclusion, PD closure is recommended after gastrectomy for gastric cancer, as the rates of PH and SPH were significantly decreased, while the procedure did not significantly increase postoperative complications.

## Data Availability

The datasets used and/or analyzed during the current study are available from the corresponding author on reasonable request.
